# Age-Specific Characteristics of Adult and Pediatric Respiratory Viral Infections: A Retrospective Single-Center Study

**DOI:** 10.3390/jcm11113197

**Published:** 2022-06-03

**Authors:** Jae Kyoon Hwang, Jae Yoon Na, Jihye Kim, Jae-Won Oh, Yong Joo Kim, Young-Jin Choi

**Affiliations:** 1 Department of Pediatrics, Hanyang University Guri Hospital, Guri 11923, Korea; jkhwang@hanyang.ac.kr (J.K.H.); jaewonoh@hanyang.ac.kr (J.-W.O.); 2 Department of Pediatrics, Hanyang University Seoul Hospital, Seoul 04763, Korea; hypedheart@hanyang.ac.kr (J.Y.N.); bluewater221@hanmail.net (J.K.); kyjoo@hanyang.ac.kr (Y.J.K.); 3 Department of Pediatrics, College of Medicine, Hanyang University, Seoul 04763, Korea

**Keywords:** age-specific characteristics, respiratory virus, viral infections, co-infection

## Abstract

This study aimed to identify age-specific characteristics of respiratory viral infections. Hospitalized patients with confirmed viral respiratory infections were included in the sample. The patients were divided into the pediatric group (<19 years old) and the adult group (≥19 years old). The groups were then subdivided based on age: 0–6, 7–12, 13–18, 19–49, 50–64, and ≥65 years old. These groups were compared to evaluate the differences in the pattern of respiratory viral infections. Among a total of 4058 pediatric patients (mean age 3.0 ± 2.9 years, *n =* 1793 females), 2829 (48.9%) had mono-infections, while 1229 (51.1%) had co-infections. Co-infections were the most common in the 0–6-year-old group (31.6%). Among 1550 adult patients (mean age 70.2 ± 15.3 years, *n =* 710 females), 1307 (85.6%) had mono-infections and 243 (14.4%) had co-infections. Co-infections were most common in the ≥65-year-old group (16.8%). Viral infection and co-infection rates decreased with age in pediatric patients but increased with increasing age in adults. In pediatric patients, the rates of viral infections and co-infections were high; the rate of co-infections was higher in younger patients. In adult patients, the rates of viral infections and co-infections were lower than those in pediatric patients; the rate of co-infections was higher in older patients.

## 1. Introduction

In recent years, concerns regarding the spread of infectious diseases have increased due to the outbreaks of H5N1 avian influenza, severe acute respiratory syndrome (SARS), and, in particular, the severe acute respiratory syndrome coronavirus 2 (SARS-CoV-2) or COVID-19 pandemic. In this context, there is growing interest in the patterns of viral infectious diseases [1,2]. Acute respiratory viral infections are the most common infectious diseases in children, accounting for 30–50% of outpatient visits and 20–40% of hospital admissions among pediatric patients [3,4,5].

Worldwide, acute respiratory viral infections kill millions of pediatric patients each year [4,6]. Recently, viruses have been reported to be more commonly detected than bacteria as the cause of pneumonia among adults; additionally, similarly to bacteria, viruses can cause severe pneumonia [7]. According to a recent prospective study, viral infections were detected in 38% of adult patients with lower respiratory tract infections, which was 11% higher than the proportion for which bacterial infections were responsible [8]. Another study revealed that, among adult patients diagnosed with viral pneumonia, the mortality rate during hospitalization amounted to 7.9% [9]. Additionally, the mortality rate due to viral infection was reported to vary from 2–17%, which is higher than that associated with bacterial pneumonia [9,10,11,12].

While respiratory viral infections are of great concern in both pediatric patients and adults, the pattern and form of disease differ between the two groups. Even within the same group, there are differences based on age [11]. In this study, we aimed to evaluate the differences in the patterns of viral infections in pediatric patients and adults. Patients admitted to a single university hospital with worsening respiratory symptoms over the past 5 years were classified by age, to investigate the differences in the characteristics between the groups. In addition, we subdivided and grouped pediatric and adult patients by age, to identify the characteristics of each group. Additionally, the distribution of the virus and the ratios of single infections and co-infections were evaluated. These findings are intended to help to identify the age-specific characteristics of viral infections and to offer advice regarding appropriate countermeasures, given the high possibility of various viral infection pandemics in the future.

## 2. Materials and Methods

### 2.1. Participant Recruitment

Patients were recruited from those who were hospitalized with respiratory symptoms, including dyspnea, cough, sputum, and fever (body temperature exceeding 38 °C), in the Hanyang University Guri Hospital from 1 January 2015 to 31 December 2019. After the COVID-19 pandemic, it was thought that analysis would be difficult because other virus infections sharply declined due to wearing masks, so only those patients hospitalized up to 2019 were recruited.

**Figure 1:**
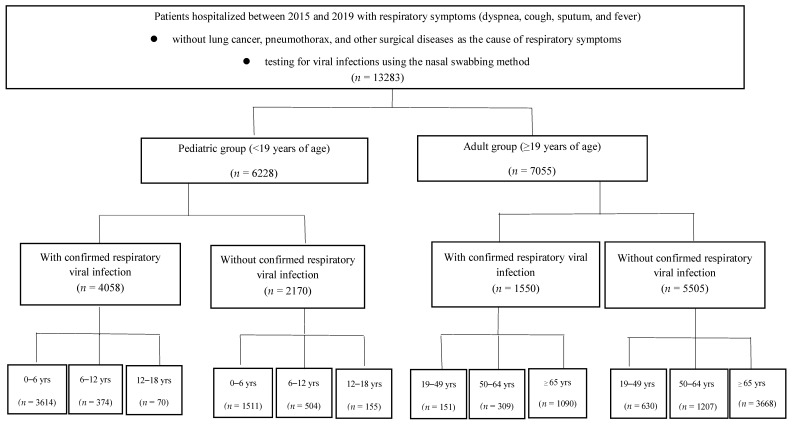
Enrollment and grouping of study patients, according to inclusion and exclusion criteria.

First, the patients were divided into a pediatric group and an adult group (≥ 19 years old). Furthermore, the pediatric and adult groups were each subdivided into three groups, based on age: 0–6, 7–12, 13–18, 19–49, 50–64, and ≥65 years old. In pediatric patients, the ages were based on the level of schooling (i.e., preschool, elementary school, and middle/high school); among adults, ages were based on the level of social activity (Figure 1).

### 2.2. Inclusion and Exclusion Criteria

Among the recruited patients, those with confirmed respiratory virus infections were selected for inclusion. Hospitalized patients with lung cancer, pneumothorax, and surgical disease as the cause of their respiratory symptoms were excluded from the sample. Additionally, patients requiring outpatient treatment for influenza of low severity were excluded, and only patients with severe influenza who were admitted to the hospital were included; therefore, the number of reported cases was lower than the actual number of infected patients (see Figure 1).

### 2.3. Viral Testing

For hospitalized patients with respiratory symptoms, testing for viral infections was performed on the first day of hospitalization, using the nasal swabbing method. Tests for 12 viruses were conducted, including adenovirus, influenza A and B viruses, human metapneumovirus (HMPV), human coronavirus (HCoV), parainfluenza virus, respiratory syncytial virus (RSV) types A and B, human rhinovirus, and human bocavirus (HBoV). All viruses were detected using a multiplex real-time (RT)-PCR assay (Allplex TM Respiratory Panel 1, 2, 3, Seegene, Seoul, Korea). The patients were diagnosed as being positive for infection if they had positive test results for at least one of the subtypes.

### 2.4. Definitions

#### 2.4.1. Viral Infection Rate

The infection rate was defined as the ratio of the total number of patients with a confirmed viral infection to the total number of patients hospitalized for respiratory symptoms. Regardless of the virus type, all cases were included in the analysis of viral infections. The number of patients hospitalized for respiratory symptoms was counted on a daily basis, and the number of confirmed viral infections was counted to determine the infection rate. The infection rate was calculated as the ratio of these two numbers.

#### 2.4.2. Co-Infection

Co-infection was defined as the confirmed presence of two or more different viruses. Viruses of different subtypes were defined as one infection.

### 2.5. Data Collection

The medical records from the charts of patients hospitalized in Hanyang University Guri Hospital from 1 January 2015 to 31 December 2019 were retrospectively reviewed. Gender, age, chief complaints at admission, underlying disease, and respiratory virus test findings of the enrolled patients were collected from the medical records.

### 2.6. Statistical Analysis

We collected and analyzed the data using an electronic spreadsheet. Descriptive statistics were used to analyze demographic data. Continuous variables were presented as mean ± standard deviation. The statistical analysis was performed using IBM SPSS Statistics, version 21.0 (IBM Co., Armonk, NY, USA).

## 3. Results

### 3.1. Demographic Characteristics

A total of 13,283 patients were hospitalized for respiratory symptoms over the 5-year study period; 7055 (mean age: 69.4 ± 15.4 years, *n =* 2760 females) and 6228 (mean age 3.6 ± 3.6 years, *n =* 2749 females) were adult and pediatric patients, respectively. Confirmed respiratory viruses were detected in 1550 adult patients (mean age 70.2 ± 15.3 years, *n =* 710 females) and 4058 pediatric patients (mean age 3.0 ± 2.9 years, *n =* 1793 females) (Table 1).

### 3.2. Types of Viruses Detected

Various respiratory viruses were identified via diagnostic testing among the hospitalized patients. In pediatric patients, the most commonly identified virus was rhinovirus (*n =* 1480), followed by adenovirus (*n =* 1113), RSV (*n =* 716), parainfluenza (*n =* 547), HboV (*n =* 545), influenza (*n =* 443), HMPV (*n =* 302), and HCoV (*n =* 250).

In adult patients, the most commonly identified virus was influenza (*n =* 435), followed by rhinovirus (*n =* 428), RSV (*n =* 185), HCoV (*n =* 183), HMPV (*n =* 152), parainfluenza (*n =* 141), adenovirus (*n =* 99), and HBoV (*n =* 16).

### 3.3. Viral Infection Rates

**Figure 2:**
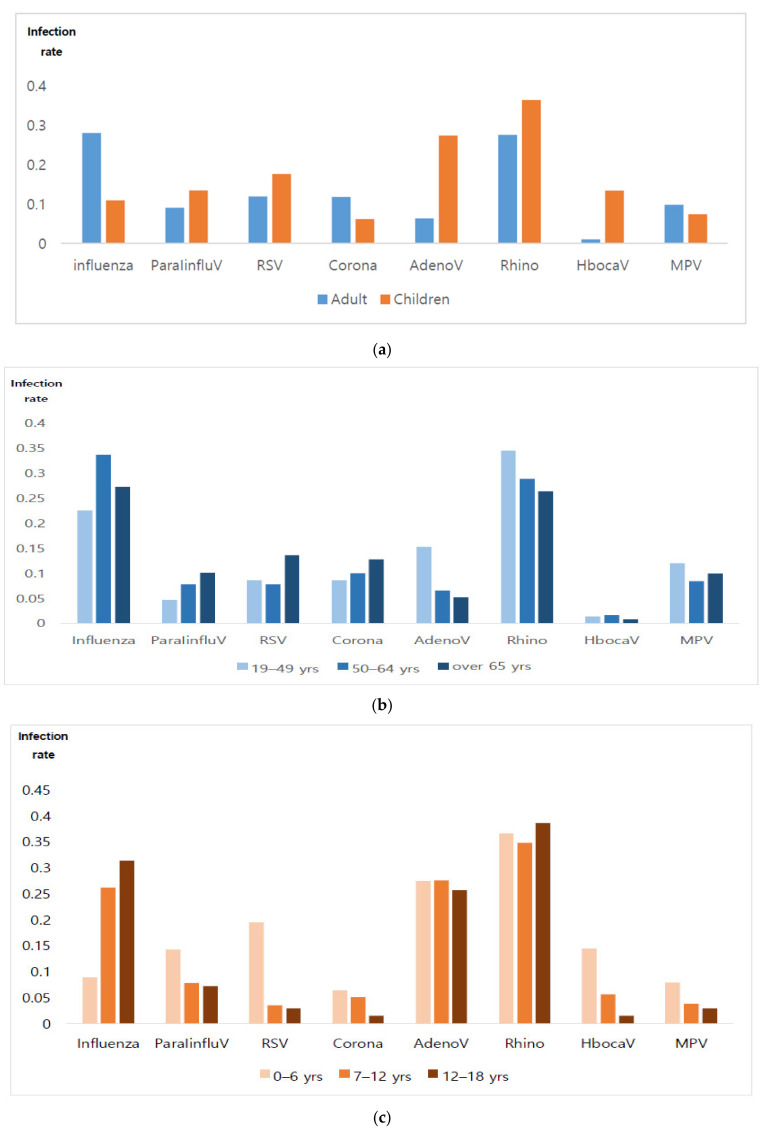
(**a**) Distribution of different types of viral infection rate detected in adult and pediatric patients. (1) The rhinovirus infection rate was highest in children. (2) The influenza infection rate was highest in adults, followed by rhinovirus infection. (**b**) Distribution of different types of viral infection rate detected, based on age categorization in adults. (1) The influenza infection rate was highest in the group of adults over 50 years, while the rhinovirus infection rate was highest in the groups of 19–49-year-olds. (**c**) Distribution of different types of viral infection rate detected, based on age categorization in pediatrics. (1) The rhinovirus infection rate was high in children of all age groups.

The distribution of viral infection rates varied between adult and pediatric patients. The rhinovirus infection rate was highest in children, while the influenza infection rate was highest in adults, followed by that of rhinovirus infection. When each group of children and adults was subdivided by age and further analyzed for comparison, there was a difference in the infection rate (see Table 2, Figure 2).

Among the pediatric patients, respiratory viral infection was confirmed in 65.2% of the cases (*n =* 4058/6228). Among them, the viral infection rate decreased with increasing age: 70.5%, 42.6%, and 31.3% in the groups of 0–6, 7–12, and 13–18 years old, respectively.

Among adult patients, respiratory viral infection was confirmed in 22.0% of cases (*n =* 1550/7055); 19.3%, 20.4%, and 22.9% were in the groups who were 19–49, 50–64, and ≥65 years old, respectively, suggesting that the viral infection rate increased with increasing age.

**Figure 3:**
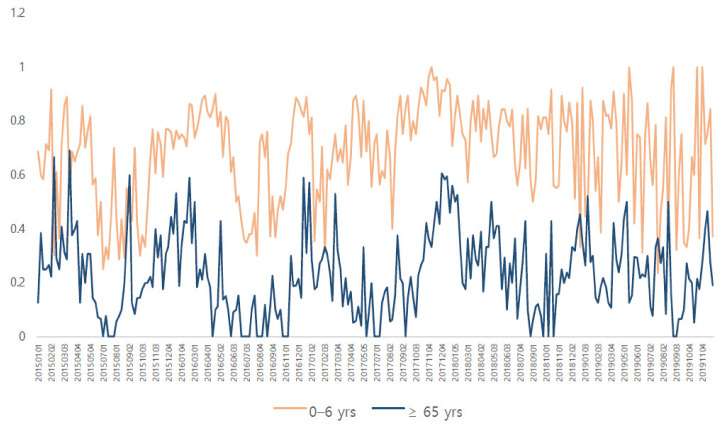
Comparison of respiratory viral infection rates in pediatric patients of 0–6 years of age and adults over 65 years of age with a high infection rate. Both graphs show a similar pattern.

When comparing pediatric patients aged 0–6 years old and adults aged ≥65 years old, there was a similar distribution shown for the viral infection rate (see Figure 3).

### 3.4. Co-Infection

Of the 4058 pediatric patients, 2829 (48.9%) had a mono-infection and 1229 (51.1%) had co-infections (two viruses: *n =* 1006; three viruses: *n =* 202; > four viruses: *n =* 21).

Co-infection was the most common in the group aged 0–6 years old (31.6%), followed by those aged 7–12 years old and those aged 13–18 years old (19.8% and 17.1%, respectively). 

Of the 1550 adult patients, 1307 (85.6%) had a mono-infection, and 243 (14.4%) had co-infections (two viruses: *n =* 227; three viruses: *n =* 16)

Co-infection was most common in individuals aged ≥65 years old (16.8%), followed by those aged 50–64 years old and 19–49 years old (12.6% and 9.3%, respectively). The infection and co-infection rates decreased with age in pediatric patients and increased with age in adult patients (see Table 3).

The most common combination of viruses in children was adenovirus + rhinovirus co-infection (*n =* 181), followed by RSV + rhinovirus co-infection (*n =* 92) and HBoV + rhinovirus co-infection (*n* = 88). The most common combination of viruses in adults was influenza + rhinovirus co-infection in patients aged over 65 years old (*n =* 21) and influenza + HCoV in those aged under 65 years old (*n =* 14).

The most common combinations of three viruses in children was of adenovirus + rhinovirus + HBoV (*n =* 37), followed by parainfluenza + rhinovirus + HboV (*n =* 16) and parainfluenza + rhinovirus + HboV (*n =* 14). The most common combination of 4 or more viruses was of parainfluenza + adenovirus + rhinovirus + HBoV.

## 4. Discussion

The results of the present study revealed differences in the patterns of respiratory viral infections between pediatric patients and adult patients. When pediatric patients and adult patients were grouped based on age, there was a difference in respiratory viral infection patterns. Among pediatric patients, the proportion of rhinovirus cases was the highest among all respiratory viral infections, while the proportion of influenza cases was the highest in adult patients. Furthermore, we found that a younger age in the adult group was associated with a higher proportion of rhinovirus cases, while older age was associated with a higher proportion of influenza cases. Even among adults, there was a difference in the proportion of the type of virus identified, based on age. 

Since our study was conducted among hospitalized patients, our results are not due to the actual difference in viral infection rates based on age. It can be thought of as the rate of infection with a virus that causes a worsening of symptoms that is severe enough to require hospitalization. In young children, a virus that is known to cause mild upper respiratory symptoms, such as rhinovirus, also causes the aggravation of respiratory symptoms and requires hospitalization. However, in adults, symptoms are exacerbated by viruses such as influenza. 

In our pediatric patient cohort, while the rate of viral infections among patients with respiratory symptoms was high, the rate of co-infections was also high; younger age was associated with a higher rate of co-infections. Among adults, the rate of viral infections among patients with respiratory symptoms was lower than that among pediatric patients and the rate of co-infections was also lower. In addition, among adults, the rate of co-infections was higher among older patients. 

These findings may be due to the nature of the respiratory tract in children and the weak respiratory protective immunity of younger children, as compared to adults and older children [13,14]. They may also be due to age-associated changes in immune responses that occur as a consequence of advanced age [15]. In addition, recent studies have demonstrated that exposure to air pollution and pollen affects viral infection [16,17]. This difference in the environment of the patients also affects the degree and type of viral infection. Previous studies showed that co-infections are as common as mono-infections in pediatric patients [18,19,20], and reported co-infection was approximately 30% in pediatric patients and 5% in adults [20,21]. Similarly, in our study, co-infections were more common than mono-infections in children [20]. However, the rate of co-infections in both pediatric patients and adult patients was higher than that reported in previous studies. These previous studies also found that the severity and hospitalization rates were higher in cases with co-infections [19,20,21]. Since we focused on hospitalized patients, only patients with high-severity illnesses were included, which may have resulted in the finding of a high co-infection rate. 

There was a greater proportion of cases with co-infection than of mono-infection in pediatric patients. The co-infection and viral infection rates were the highest among those aged 0–6 years old. This result may be due to the fact that such patients are frequently exposed to various types of viruses due to exposure to other children in daycare and/or kindergarten. However, among adults, the co-infection rate was higher in the elderly, who are less active and are less likely to come into contact with other infected individuals. This finding suggests that co-infection is affected not only by the frequency of contact or the diversity of contact but also by the patient’s overall health or immune status. Previous studies reported that virus co-infection rates increase when receiving immunosuppressive therapy [19]. In addition, it was found that various factors affect co-infections. Co-infections may be determined based on the type of virus [21]. Adenovirus was reported to have a surprisingly high prevalence among the different types of co-infections, especially in pediatric patients, while influenza virus B had the lowest prevalence [20,21]. 

Evaluating the age-dependence of different viral infections will be helpful in suggesting different countermeasures for patients with respiratory symptoms according to age. Therefore, further large-scale, well-characterized cohort studies are needed to demonstrate a more accurate age-dependence characteristic for different viral infection rates.

The present study has several limitations. First, as our research was a single-center study, there are regional and time limitations. Second, we analyzed a limited number of cases, as only patients with severe illness who required hospitalization were included in the sample. Third, it is not clear whether the detected virus actually acted as a pathogen or was a virus that colonized the upper respiratory tract. In one study of healthy children, the intranasal virus positivity rate was reported as 40% for rhinovirus, 20% for enterovirus, 10% for bocavirus, and 5% for adenovirus [22]. Therefore, there is a compelling need to perform multi-center studies with common protocols, which would last several years and would measure the viral load for different viruses in co-infections. Comparisons of viral detection in a control group with asymptomatic children may help to better understand which viruses can be innocent rhinopharyngeal colonizers.

Despite these limitations, our results confirmed that there are age-specific characteristics in viral infection among hospitalized patients with respiratory symptoms. While previous studies revealed the difference in viral infection between children and adults, few of those studies grouped children or adults by age and compared them. Our attempt to do so has confirmed that there are meaningful differences in the virus infection patterns within child and adult groups.

There is evidence that people with underlying diseases such as heart disease, chronic lung disease, malignancy, diabetes, and chronic kidney disease can die from fatal respiratory complications [10,23]. Respiratory virus infections can rapidly spread within communities and can cause serious social and economic losses, as has been observed as a result of influenza, SARS, and, particularly, the recent COVID-19 pandemic. Therefore, it is important to understand the patterns of viral infections. The results of the present study can help to understand the characteristics of age-specific viral infections, although a more extensive study would be necessary to clarify this issue.

## 5. Conclusions

In this study, we found differences in the patterns of respiratory viral infections between pediatric and adult patients. In pediatric patients, the rate of viral infections among those with respiratory symptoms was high, as was the rate of co-infections. Younger age was found to be associated with a higher rate of co-infection in pediatric patients. Among adults, the rate of viral infections among those with respiratory symptoms was lower than that among pediatric patients, as was the rate of co-infections. Older age was found to be associated with a higher rate of co-infection in adult patients. These characteristics of age-specific viral infections provide important clinical implications for virus prevention and treatment.

## Figures and Tables

**Table 1:** Demographic characteristics.

	N	Age, yrs	Sex (M:F)
Total pediatric patients	6228	3.6 ± 3.6	3479:2749
0–6 years old	5125	2.2 ± 1.8	2869:2256
6–12 years old	878	9.0 ± 1.6	500:378
12–18 years old	225	14.6 ± 1.6	110:115
Pediatric patients infected	4058	3.0 ± 2.9	2265:1793
0–6 years old	3614	2.2 ± 1.6	2010:1604
6–12 years old	374	8.7 ± 1.6	217:157
12–18 years old	70	14.2 ± 1.2	38:32
Total adult patients	7055	69.4 ± 15.4	4295:2760
19–49 years old	781	38.0 ± 9.2	461:140
50–64 years old	1516	57.9 ± 4.1	975:541
≥65 years old	4758	78.1 ± 7.1	2859:1899
Adult patients infected	1550	70.2 ± 15.3	840:710
19–49 years old	151	36.6 ± 9.7	84:67
50–64 years old	309	57.8 ± 4.0	163:146
≥65 years old	1090	78.3 ± 7.2	593:497

Note. Data are presented as numbers or means ± standard deviation.

**Table 2:** Distribution of different types of viral infection rates detected in adult and pediatric patients, according to age.

	Virus Infection Rate					
	0–6 yrs	7–12 yrs	12–18 yrs	19–49 yrs	50–64 yrs	≥65 yrs
Influenza	0.09	0.26	0.31	0.23	**0.34**	**0.27**
ParaIinfluV	0.14	0.08	0.07	0.05	0.08	0.10
RSV	0.19	0.03	0.03	0.09	0.08	0.14
Corona	0.06	0.05	0.02	0.09	0.10	0.13
Adeno	0.27	0.28	0.26	0.15	0.07	0.05
Rhino	**0.37**	**0.35**	**0.39**	**0.35**	0.29	0.26
Hboca	0.14	0.06	0.01	0.01	0.02	0.01
MPV	0.08	0.04	0.03	0.12	0.08	0.10

Note. Bold font denotes the highest infection rate.

**Table 3:** Virus infection patterns (infection rate, co-infection rate).

	Infection Rate	1 Virus Infected	2 Viruses Co-Infected	3 Viruses Co-Infected	≥4 Viruses Co-Infected
Pediatric patients	4058/6228 (65.2%)	2829 (48.9%)	1006 (17.4%)	202 (3.5%)	21 (0.3%)
0–6 years	3614/5125 (70.5%)	2471 (68.4%)	929 (25.7%)	193 (5.3%)	21 (0.6%)
7–12 years	374/878 (42.6%)	300 (80.2%)	65 (17.4%)	9 (2.4%)	0
13–18 years	70/225 (31.1%)	58 (82.9%)	12 (17.1%)	0	0
Adult patients	1550/7055 (22.0%)	1304 (84.1%)	237 (15.3%)	19 (1.2%)	0 (0%)
19–49 years	151/781 (19.3%)	137 (90.7%)	9 (6.0%)	5 (3.3%)	0
50–64 years	309/1516 (20.4%)	260 (84.1%)	47 (15.2%)	2 (0.6%)	0
≥65 years	1090/4758 (22.9%)	907 (85.3%)	181 (16.6%)	12 (1.1%)	0

Note: Data are presented as numbers and proportions.

## Data Availability

The data presented in this study are available on request from the corresponding author. The data are not publicly available due to restrictions e.g., privacy or ethical.

## References

[1] Wu Z., McGoogan J.M. (2020). Characteristics of and important lessons from the coronavirus Disease 2019 (COVID-19) outbreak in China: Summary of a report of 72,314 cases from the Chinese Center for Disease Control and Prevention. JAMA.

[2] Marroquín B., Vine V., Morgan R. (2020). Mental health during the COVID-19 pandemic: Effects of stay-at-home policies, social distancing behavior, and social resources. Psychiatry Res..

[3] Kim M.R., Lee H.R., Lee G.M. (2000). Epidemiology of acute viral respiratory tract infections in Korean children. J. Infect..

[4] Guerrier G., Goyet S., Chheng E.T., Rammaert B., Borand L., Te V., Try P.L., Sareth R., Cavailler P., Mayaud C. (2013). Acute viral lower respiratory tract infections in Cambodian children: Clinical and epidemiologic characteristics. Pediatr. Infect. Dis. J..

[5] (2018). GBD 2016 Lower Respiratory Infections Collaborators. Estimates of the global, regional, and national morbidity, mortality, and aetiologies of lower respiratory infections in 195 countries, 1990–2016: A systematic analysis for the Global Burden of Disease Study 2016. Lancet Infect. Dis..

[6] Yu J., Xie Z., Zhang T., Lu Y., Fan H., Yang D., Bénet T., Vanhems P., Shen K., Huang F. (2018). Comparison of the prevalence of respiratory viruses in patients with acute respiratory infections at different hospital settings in North China, 2012–2015. BMC Infect. Dis..

[7] Jain S., Self W.H., Wunderink R.G., Fakhran S., Balk R., Bramley A.M., Chappell J.D. (2015). Community-acquired pneumonia requiring hospitalization among US adults. N. Engl. J. Med..

[8] Ieven M., Coenen S., Loens K., Lammens C., Coenjaerts F., Vanderstraeten A., Henriques-Normark B., Crook D., Huygen K., Butler C. (2018). Aetiology of lower respiratory tract infection in adults in primary care: A prospective study in 11 European countries. Clin. Microbiol. Infect..

[9] Lee N., Lui C.Y.G., Wong K.T., Li T.C.M., Tse E.C.M., Chan J.Y.C., Yu J., Wong S.S.M., Choi K.W., Wong R.Y.K. (2013). High morbidity and mortality in adults hospitalized for respiratory syncytial virus infections. Clin. Infect. Dis..

[10] Falsey A.R., Hennessey P.A., Formica M.A., Cox C., Walsh E.E. (2005). Respiratory syncytial virus infection in elderly and high-risk adults. N. Engl. J. Med..

[11] Katsurada N., Suzuki M., Aoshima M., Yaegashi M., Ishifuji T., Asoh N., Hamashige N., Abe M., Ariyoshi K. (2017). The impact of virus infections on pneumonia mortality is complex in adults: A prospective multicentre observational study. BMC Infect. Dis..

[12] Kwon Y.S., Park S.H., Kim M.-A., Kim H.J., Park J.S., Lee M.Y., Lee C.W., Dauti S., Choi W.-I. (2017). Risk of mortality associated with respiratory syncytial virus and influenza infection in adults. BMC Infect. Dis..

[13] Simon M., Collins M.S. (2013). The pediatric lung and aspiration. Perspect. Swallowing Swallowing Disord..

[14] David R., Tohomas M. (2018). Overview of innate lung immunity and inflammmation. Lung Innate Immunity and Inflammation.

[15] Keith M. (2010). The Role of Immunity and Inflammation in Lung Senescence and Susceptibility to Infection in the Elderly. Semin. Respir. Crit. Care Med..

[16] Choi Y., Lee K., Lee Y., Kim K., Oh J. (2022). Analysis of the association among air pollutants, allergenic pollen, and respiratory virus infection of children in Guri, Korea during recent 5 years. Allergy Asthma Immunol. Res..

[17] Annesi I., Maesano C.N., D’Amato M., D’Amato G. (2021). Pros and cons for the role of air pollution on COVID-19 development. Allergy.

[18] Visseaux B., Collin G., Ichou H., Charpentier C., Bendhafer S., Dumitrescu M., Allal L., Cojocaru B., Desfrère L., Descamps D. (2017). Usefulness of multiplex PCR methods and respiratory viruses’ distribution in children below 15 years old according to age, seasons and clinical units in France: A 3 years retrospective study. PLoS ONE.

[19] Stefanska I., Romanowska M., Donevski S., Gawryluk D., Brydak L.B. (2013). Co-infections with influenza and other respiratory viruses. Respiratory Regulation-The Molecular Approach.

[20] Mandelia Y., Procop G., Richter S., Worley S., Liu W., Esper F. (2021). Dynamics and predisposition of respiratory viral co-infections in children and adults. Clin. Microbiol. Infect..

[21] Ching N.S., Kotsanas D., Easton M.L., Francis M.J., Korman T.M., Buttery J.P. (2018). Respiratory virus detection and co-infection in children and adults in a large Australian hospital in 2009–2015. J. Paediatr. Child. Health.

[22] van den Bergh M.R., Biesbroek G., Rossen J.W.A., de Steenhuijsen Piters W.A.A., Bosch A.A.T.M., van Gils E.J.M., Wang X., Boonacker C.W., Veenhoven R.H., Bruin J.P. (2012). Associations between pathogens in the upper respiratory tract of young children: Interplay between viruses and bacteria. PLoS ONE.

[23] Glezen W.P. (1993). Influenza surveillance in an urban area. Can. J. Infect. Dis..

